# simGWAS: a fast method for simulation of large scale case–control GWAS summary statistics

**DOI:** 10.1093/bioinformatics/bty898

**Published:** 2018-10-29

**Authors:** Mary D Fortune, Chris Wallace

**Affiliations:** 1 MRC Biostatistics Unit, Cambridge Institute of Public Health, University of Cambridge, Cambridge Biomedical Campus, Cambridge, UK; 2 Department of Medicine, University of Cambridge, Addenbrooke’s Hospital, Cambridge, UK

## Abstract

**Motivation:**

Methods for analysis of GWAS summary statistics have encouraged data sharing and democratized the analysis of different diseases. Ideal validation for such methods is application to simulated data, where some ‘truth’ is known. As GWAS increase in size, so does the computational complexity of such evaluations; standard practice repeatedly simulates and analyses genotype data for all individuals in an example study.

**Results:**

We have developed a novel method based on an alternative approach, directly simulating GWAS summary data, without individual data as an intermediate step. We mathematically derive the expected statistics for any set of causal variants and their effect sizes, conditional upon control haplotype frequencies (available from public reference datasets). Simulation of GWAS summary output can be conducted independently of sample size by simulating random variates about these expected values. Across a range of scenarios, our method, produces very similar output to that from simulating individual genotypes with a substantial gain in speed even for modest sample sizes. Fast simulation of GWAS summary statistics will enable more complete and rapid evaluation of summary statistic methods as well as opening new potential avenues of research in fine mapping and gene set enrichment analysis.

**Availability and implementation:**

Our method is available under a GPL license as an R package from http://github.com/chr1swallace/simGWAS.

**Supplementary information:**

Supplementary data are available at *Bioinformatics* online.

## 1 Introduction

The genome wide association study design is now more than a decade old (Visscher *et al.*, 2017), and the size of GWAS cohorts has continued to grow, from 1000 s to, now, 1 000 000 s of individuals. Given the competing demands of open science and privacy concerns (P3G Consortium *et al.*, 2009), it has become standard to share data in the form of summary statistics (allelic effect sizes and standard errors, or simply *P* values) more readily than the full genotype data. A wealth of methods have been developed to operate directly on the summary statistics, from fine mapping of genetic causal variants [e.g. PAINTOR (Kichaev *et al.*, 2014), CAVIARBF (Chen *et al.*, 2015) and JAM (Newcombe *et al.*, 2016)] to (co-)heritability estimation (Bulik-Sullivan *et al.*, 2015) and integration of GWAS results from different traits (Giambartolomei *et al.*, 2014; Zhu *et al.*, 2016). Summary data methods are often derived through approximating a multivariate linear regression likelihood by incorporating information about correlation structures (linkage disequilibrium, LD) from reference populations. However, one must adopt a logistic regression approach to correctly model risk on the log odds scale when analyzing GWAS of binary traits (including case–control data). Summary statistic methods which have been originally derived for linear regression cannot do this and the impact of the linearity assumption on their conclusions if applied to case–control data has not been investigated in depth.

As Biobank-sized datasets come to fruition, such summary statistic methods are likely to become even more important, since, for such large numbers of samples, operating on the complete genotype data matrices for efforts such as Bayesian fine mapping of causal variants is computationally prohibitive. Indeed, GWAS summary statistics for multiple traits from UK-Biobank have already been made freely available (Canela-Xandri *et al.*, 2018). While Biobanks tend to adopt a cohort design, meta-GWAS studies continue to over-sample cases compared to controls, in order to increase the available power, and are now exceeding 100 000 cases and controls in single studies (Michailidou *et al.*, 2017).

The gold standard for evaluating performance of summary statistic methods is through analysis of simulated data, allowing inference to be compared to a known ‘truth‘. A common method used by GWAS simulators is to proceed by adding phenotypes to a sample of genotype data that is either simulated or from a reference population (‘forward simulation‘). This approach, in particular, can be used very flexibly for generating multiple (quantitative) phenotypes, a design also common to Biobank datasets (Meyer and Birney, 2018). However, this method does not lend itself to simulating case–control data, since it simulates cases in proportion to what we would expect to see in the reference population; typical GWAS designs recruit cases disproportionally to their frequency in the population in order to increase power. In order to forward simulate a GWAS cohort, we would need to simulate until we had the required number of cases and controls, discarding additional samples (typically a large number of controls as cases are normally a minority in the population). This is computationally expensive, and wasteful.

Instead, when simulating case–control data, we typically simulate or sample genotype data conditional on a supposed distribution of phenotypes. Simulation options in this case are more limited because the problem is mathematically harder. For single causal variant scenarios, resampling from a reference population conditional on allele frequencies at a target variant may be used. For more complicated causal models, involving multiple variants potentially in LD, GWAsimulator (Li and Li, 2008), TriadSim (Shi *et al.*, 2018) or HAPGEN (Su *et al.*, 2011) can very efficiently simulate haplotypes for cases and controls in small genomic regions. In particular, by incorporating mutations and recombinations, HAPGEN can simulate large populations with only a few hundred reference haplotypes. However, the generation of GWAS summary statistics, e.g. using SNPTEST (Marchini *et al.*, 2007), requires analysis of the individual level data which can be slow, particularly for logistic models which require iterative optimization at each SNP.

The general approach of simulating both genotype and phenotype on an individual level cannot scale well for Biobank-scale or large meta-GWAS situations, because of the number of individuals required. It is also potentially wasteful—the individual level data are not required when the goal is to evaluate methods that work on summary statistics.

Here, we present an alternative approach, which simulates summary statistics directly, without needing to ever generate genotype data. It scales as a function of the number of SNPs, but is constant with regards to the number of samples, thus making it ideally designed for simulation of summary statistics for large case–control studies.

## 2 Materials and methods

### 2.1 Overview of our approach

We first introduce the mathematical calculations which underpin our method. Given a causal model specifying a region of interest, which SNPs in the region are causal, their effects on disease in the form of odds ratios, and reference data on allele and haplotype frequencies in controls, we calculate the expected *Z* score from a Cochran Armitage score test under an additive model at each SNP in the region. [Cochran Armitage score tests have been used for GWAS because of their computational simplicity, requiring no iterative maximization procedure, and because they allow for additive, dominant or recessive coding, although additive coding is the most commonly used (Sasieni, 1997)].

Simulated Z scores can then be derived by multivariate normal simulation using standard software, with the variance-covariance matrix calculated from correlations between the SNPs in the reference data. This suffices in the case where the summary statistic methods to be used work upon Z scores alone. However, when log odds ratios and their standard errors are required, we appeal to the asymptotic similarity of score tests and Wald tests, and simulate standard errors under the causal model. Together with simulated Z scores, we can then back-calculate the log odds ratios as the product of simulated Z scores and standard errors. An outline description of our calculations follows; full details are given in the Supplementary Material.

Let $Y_{i}\in \{0,1\}$ denote the indicator of disease status for the *i*th of *N* individuals sampled according to case–control status (*N*_1_ cases, *Y _i_* = 1; *N*_0_ controls, *Y _i_* = 0). Let *n* be the total number of SNPs. For any SNP *X*, write $G_{i}^{X}$ for its genotype coding ∈{0,1,2} at sample *i.* Then, for the commonly used Cochran-Armitage score test, the Z-Score at SNP *X* is computed as:

$$Z_{X}=\frac{\sum_{i=1}^{N}((G_{i}^{X}-\overset{¯}{G^{X}})(Y_{i}-\overset{¯}{Y}))}{\sqrt{\frac{N_{0}N_{1}}{N(N-1)}\sum_{i=1}^{N}{\left(G_{i}^{X}-\overset{¯}{G^{X}}\right)}^{2}}}=\frac{U_{X}}{\sqrt{(N-1)V_{X}V_{Y}}}$$

where *V_X_*, *V_Y_* denote var(X), var(Y), respectively.

Write $W={\left(W_{1},...,W_{m}\right)}^{T}$ for the vector of causal SNPs and $\gamma ={\left(\gamma_{1},...,\gamma_{m}\right)}^{T}$ for their log odds ratios of effect. We assume that *Y_i_* given $G_{i}^{W}$ can be modelled as a binomial logistic regression:

$$P_{sam}(Y_{i}=1|G_{i}^{W}=w)=\frac{e^{\gamma_{0}+\gamma_{1}w_{1}+...+\gamma_{m}w_{m}}}{1+e^{\gamma_{0}+\gamma_{1}w_{1}+...+\gamma_{m}w_{m}}}$$

where $P_{sam}()$ denotes that this is the probability within the GWAS sample and *γ*_0_ is chosen such that $P_{sam}(Y_{i}=1)=\frac{N_{1}}{N}$. The conditioning is required because allele frequencies vary between cases and controls at causal variants and those in LD with them, meaning the overall allele frequencies in our sample differ from those in the population as a whole. By specifically distinguishing between $P_{sam}()$ and the more general P(), we can condition on having chosen *N*_0_ controls and *N*_1_ cases and thus perform the conditional simulation needed for case–control studies.

By conditioning upon the values of $G^{W}$ and *Y*, we obtain the expected value of *U_X_*, the covariance between *G^X^* and *Y*:

(1) E(UX)=(N−1)N0N1N2∑w∈Z3m[(N0N1eγ0+γ1w1+...+γmwm−1)] ×[2P(GiX=2∩GiW=w)+P(GiX=1∩GiW=w)]

The variance of *U_X_* is $V_{X}V_{Y}$ where $V_{Y}=\frac{N_{0}N_{1}}{N(N-1)}$ and *V_X_* is the variance of *G^X^.* As *V_X_* is a variance, a natural model is an inverse gamma distribution, $V_{X}∼\Gamma^{-1}(\alpha ,\beta )$. By similar conditioning upon $G^{W}$ and *Y*, we show that the parameters of this distribution are

(2) $$\alpha =\frac{2E(V_{X}^{2})-{\left(E(V_{X})\right)}^{2}}{E(V_{X}^{2})-{\left(E(V_{X})\right)}^{2}}$$

(3) $$\beta =\frac{E(V_{X})E(V_{X}^{2})}{E(V_{X}^{2})-{\left(E(V_{X})\right)}^{2}}$$

(the derivation of this and expressions for the first two moments of *V_X_* are given in the Supplementary Material). This means we can either simulate *V_X_* from its distribution or calculate

$$E\left(\frac{1}{\sqrt{V_{X}}}\right)=\frac{1}{\sqrt{\beta }}\frac{\Gamma \left(\frac{2\alpha +1}{2}\right)}{\Gamma (\alpha )}$$

so that, to a first order approximation,

$$E(Z_{X})\approx E(U_{X})\times E\left(\frac{1}{\sqrt{V_{X}}}\right)\times \sqrt{\frac{N_{0}N_{1}}{N(N-1)}}$$

Putting this together, we can now calculate the expected *Z* score, $Z^{\text{E}}$, across a set of SNPs, given a causal model and some phased reference data with which to calculate the probabilities in (Equation 1). Note that the computational complexity of this calculation is independent of both disease frequency and the number of samples required.

For some applications, the expected Z Score may suffice. However, note that the expected GWAS *P* value is not the *P* value associated with the expected Z score. Instead, we must simulated ‘observed‘ GWAS results which vary randomly about $Z^{\text{E}}$, with variance 1, such that the correlation between the *Z* score at two SNPs is equal to the correlation between their genotypes (Burren *et al.*, 2014). It is hence computationally simple to simulate multiple realizations of GWAS *Z* scores as $Z^{\text{*}}∼\text{MVN}(Z^{\text{E}},\Sigma )$, where Σ is a matrix describing correlation between SNPs for the region, again estimated from the reference panel.

To generate log odds ratios, *γ*, and their standard errors, *σ*, we appeal to the asymptotic similarity of Wald tests from a logistic regression model to the Cochran Armitage score test, and the result that the variance of the score statistic *U_X_* is the inverse of the variance of the estimated *γ*, under the null (McCullagh and Nelder, 1983). Thus, we simulate $V_{X}^{*}∼\text{Inverse Gamma}(\alpha ,\beta )$ with (α,β) given by (Equations 2, 3) and hence $V{\left(U_{X}\right)}^{*}=V_{X}^{*}V_{Y}$. Finally, we set $\sigma^{*}=\sqrt{1/V{\left(U_{X}\right)}^{*}}$ and calculate $\gamma^{*}=\sigma^{*}Z^{\text{*}}$.

### 2.2 Simulations to validate summary statistics

We evaluated our proposed method by simulating summary statistics in parallel using simGWAS (our method) and the same settings with HAPGEN2 + SNPTEST2, using reference data from 1000 Genomes Phase 3 (1000 Genomes Project Consortium *et al.*, 2015) (AFR cohort, ∼600 subjects). Reference data was downloaded from https://mathgen.stats.ox.ac.uk/impute/impute_v2.html#reference. We compared distributions of summary statistics visually and with Kolmogorov-Smirnov tests, as well as time to create the statistics under different scenarios. Full code to run these simulations is available from http://github.com/chr1swallace/simgwas-paper.

## 3 Results

### 3.1 Validation of simulated summary statistics

**Fig. 1.**
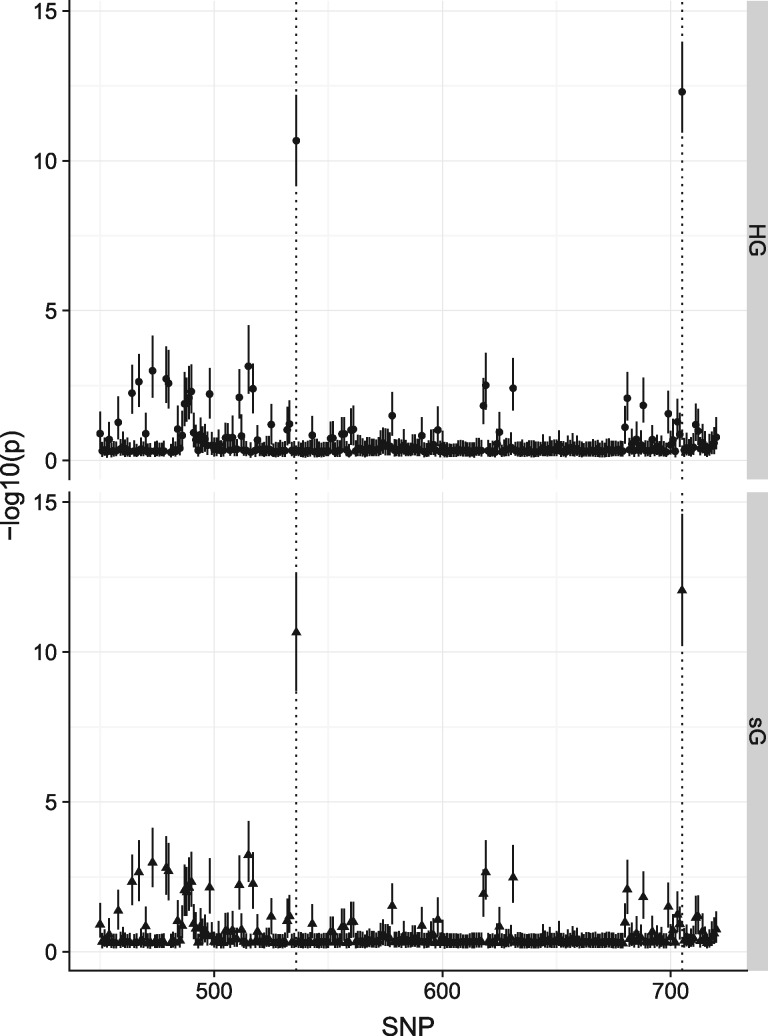
Results from simGWAS (sG) are visually similar to those from HAPGEN+SNPTEST (HG). The figure shows ∼350 SNPs from around the causal variants in the simulated region under scenario 4, with 5000 cases and 5000 controls. Points show the median −log10(*P* value) for each SNP, and ranges the IQR across 1000 simulations. Location of causal variants are marked with dotted lines

We visually confirmed that the calculated $Z^{\text{E}}$ appeared sensible for a selection of one to four independent causal SNP models in a single region (Supplementary Fig. S1). We next simulated data using our method or an individual-based method (HAPGEN+SNPTEST) for five scenarios (Table 1) for a more detailed evaluation. Note in particular the difference between scenarios 4 and 5. In scenario 4, two variants in weak LD each have a log odds ratio of log⁡(1.2)=0.18 or log⁡(1/1.2)=−0.18. In this case, marginal estimates of odds ratios are close to these values, and Z scores are highly significant. In scenario 5, the pair of odds ratios are the same, but at strongly linked variants ($r^{2}=0.8$). This would be expected to cause the effect of one to be ‘cancelled‘ by the other in the marginal associations, so that estimates of log OR are attenuated towards 1 and significance is dramatically lower, as seen for both HAPGEN+SNPTEST and simGWAS simulations (Supplementary Fig. S2). Visually, the Manhattan plots generated by the two methods were similar (Fig. 1). However, we did notice that simGWAS displayed greater variability than HapGen+SNPTEST at the SNPs with smallest *P* values. Formal comparison of the distribution of statistics showed that the mean log OR and mean Z score were statistically indistinguishable between the two methods, but that simGWAS produced results with greater variability, resulting in some statistically significant differences in Kolmogorov-Smirnov tests comparing the two distributions (Supplementary Table S1).

**Table 1.** Five simulation scenarios considered for validation of results

Scenario	Description
1	Single common causal variant, weak effect
	MAF = 0.5; odds ratio = 1.1
2	Single low frequency causal variant, strong effect
	MAF = 0.02; odds ratio = 1.5
3	Three causal variants, unlinked
	MAF = 0.27, 0.37, 0.26; odds ratios 1.1, 1.2, 1.3
4	Two causal variants, weakly linked
	r = 0.15; MAF = 0.39, 0.25; odds ratios 1.2 and 1/1.2
5	Two causal variants, strongly linked
	r = 0.8; MAF = 0.1, 0.15; odds ratios 1.2 and 1/1.2

**Fig. 2.**
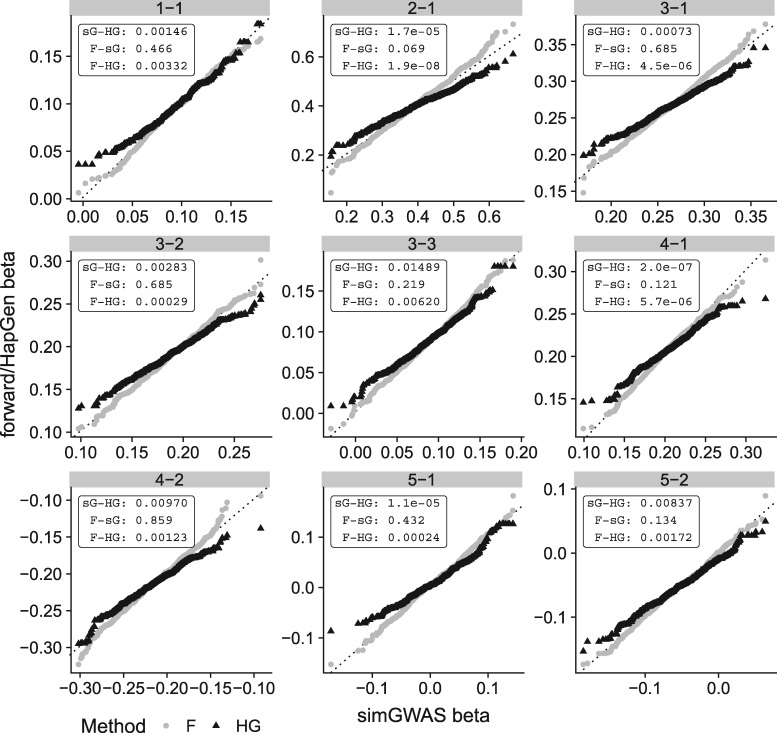
QQ plots comparing distribution of log OR at causal SNPs across 1000 simulations with 5000 cases and 5000 controls. Each plot compares the distribution of log OR generated by simGWAS (sG, *x*-axis) to that from HapGen+SNPTEST (HG) or forward simulation (F). Distributions were compared using Kolmogorov-Smirnov, and *P* values are shown in the top-right of each subplot. The label of each plot gives the corresponding ‘scenario-snp’ pair—i.e. the label 3-1 refers to scenario 3, first causal SNP

To investigate this, we conducted forward simulations at the causal variants only in each scenario, as a gold standard, and found that results from simGWAS more closely matched those from this gold standard than did those from HapGen+SNPTEST (Fig. 2, Supplementary Table S1).

**Fig. 3.**
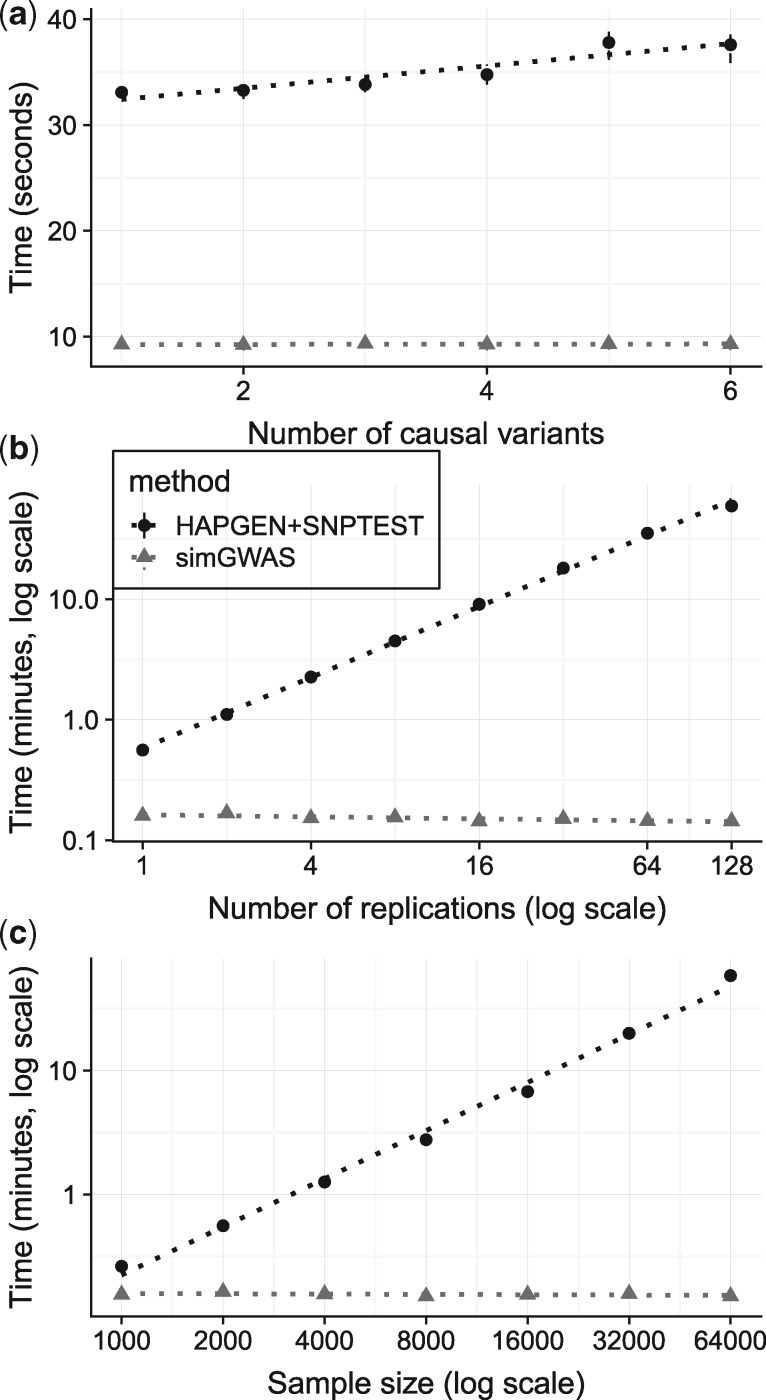
Time taken to perform simulations under simGWAS or HAPGEN2+SNPTEST2 strategies. simGWAS is denoted by triangles and HAPGEN+SNPTEST2 by circles. Each point represents the mean time for 50 independent runs of the software, with standard deviation about that mean indicated by the vertical bars. (**a**) The effect of number of causal variants on run time. 2000 cases, 2000 controls, single replication, causal variants varying from 1 to 6. (**b**) The effect of number of replications on run time. 2000 cases, 2000 controls, 2 causal variants, number of replications varying from 1 to 100. (**c**) The effect of sample size on run time. single replication, 3 causal variants, number of cases and controls (each) varying from 1000 to 64 000

Finally, we compared simulation speed of each strategy as the number of causal variants, the number of samples and the number of replicates varied. For a region with 1000 SNPs using AFR data from 1000 Genomes (∼600 samples), both methods were very fast (<30 s) for the simplest scenario of 1000 cases and 1000 controls. We found that both methods required slightly, but negligibly, more time as the number of causal variants increased from one to six (Fig. 3a). As expected, HAPGEN+SNPTEST scaled linearly with either the number of replications (number of complete sets of data simulated from the same scenarios) or sample size, whereas simGWAS timings were independent of either factor (Fig. 3b and c). This emphasizes the potential for fast simulation of summary statistics for very large case–control datasets.

## 4 Discussion

Simulating GWAS summary statistics in the context of case–control studies, for any required causal model and set of odds ratios, has several potential applications. Primarily, simulated GWAS results have become the accepted gold standard for validating newly developed statistical models for the analysis of GWAS data. Our intent is to enable the faster simulation of summary statistics compared to individual level data simulation, while at the same time using considerably less disk space. Although the method focuses on region-level simulation, it can be used to generate genomewide statistics if required, by breaking the each chromosome into approximately independent blocks, according to recombination hotspots or breakpoints derived from examining correlation between genotypes (Berisa and Pickrell, 2016).

We note that our method depends on an assumption of additivity—across alleles at each SNP, and across causal SNPs in a region. This additive model is used by the overwhelming majority of GWAS analyses, by genetic risk score approaches and by LD regression methods (Bulik-Sullivan *et al.*, 2015; Dudbridge *et al.*, 2018). Thus it seemed the sensible place to start. However, examining how these methods which assume additivity perform when the underlying model is *not* additive is an interesting research question. A future direction to extend our method could be to adapt it to simulate data under any genetic model by expressing disease risk as a function of genotypes at causal SNP haplotypes. While we have focused on retrospective case–control designs as an obvious gap in the GWAS simulation toolbox, our methodology could be relevant in the area of extreme-sampling designs, where power is maximized for a fixed cost by sampling individuals with extreme values of a quantitative trait, for example in a study of blood pressure (Warren *et al.*, 2017). We could adapt our method to this design by expressing the distribution of haplotype frequencies as a function of a quantitative trait.

In addition to supporting method development, simulation of GWAS statistics is also used in tests aggregating information across sets of SNPs, e.g. for pathway analysis. Pathway analysis can test either the *global null*, of no association between any SNP and phenotype, or the *competitive null*, which assumes there are some truly associated SNPs, but that these are randomly distributed amongst the sets of SNPs considered (i.e. those near genes in or out of the pathway under test, or those corresponding to presence or absence of a feature of interest). The second seems more appropriate, because it acknowledges that enrichment tests are performed in the context of genome-wide significant associations having been already found. However, the second is also much harder to simulate.

A common technique for simulating under a competitive null is permutation testing; the underlying dataset is maintained, and labels are permuted to generate new datasets where traits are still associated, but there is no possible correlation to the feature of interest. However, doing this so as not to destroy the genomic structure within the region, can require inventive generation of null distributions, for example, by circularization and permutation of genomic features to allow empirical null distributions to be calculated under a competitive null (Trynka *et al.*, 2015). While these are efficient, they can only be used for features that span shorter distances than LD—e.g. for chromatin mark enrichment but not genes collected in pathways.

To allow more simple simulation techniques to be used, pathway-based tests of the competitive null have been adapted to have the same expected null distribution as tests of the global null. This requires replacing *P* values for individual genes by their ranks (Evangelou *et al.*, 2012) which loses distributional information.

There is therefore potential to further develop pathway or enrichment test methodology if the distribution of test statistics under a competitive null hypothesis could be derived. Our method would naturally allow simulation of GWAS summary data under a specific hypothesis about the location and magnitude of genetic effects, in order to generate empirical null distributions for tests of the competitive null, preserving genomic structure even when analysis is performed across multiple regions.

Finally, our method could be used to evaluate output of fine-mapping applied to real data. Particularly in regions where the patterns of LD between putative associated SNPs are complex, it can be hard to dissect what the true causal variants are. Different fine mapping methods make different assumptions about the number and independence of causal variants, which can produce conflicting results (Newcombe *et al.*, 2016; Wallace *et al.*, 2015). By generating expected summary statistics under alternative fine-mapped solutions, it may be possible to see whether one or another is more compatible with observed data.

Our method enables faster simulation of GWAS case–control summary statistics compared to individual level data simulation, at the same time using considerably less disk space. This should facilitate computationally simpler evaluation of existing and new summary GWAS methods and has the potential to underpin new method development in other areas.

## Supplementary Material

bty898_Supplementary_Information

bty898_Supplementary_Table
