# Supplementary material for: simGWAS: a fast method for simulation of large scale case–control GWAS summary statistics
Source: Bioinformatics. 2018 Oct 29;35(11):1901–6. doi: 10.1093/bioinformatics/bty898 (PMC6546134; doi:10.1093/bioinformatics/bty898)
Supplement: bty898_Supplementary_Information [file bty898_supplementary_information.pdf]

Supplementary Information - simGWAS: a fast method for simulation of large scale case-control GWAS summary statistics

Mary D. Fortune[1,2] and Chris Wallace[1,2]

September 11, 2018

1. MRC Biostatistics Unit, University of Cambridge, Cambridge Institute of Public Health, Forvie Site, Robinson Way, Cambridge Biomedical Campus, Cambridge, CB2 0SR, United Kingdom

2. Department of Medicine, University of Cambridge, Addenbrooke's Hospital, Hills Rd, Cambridge CB2 0SP

Contents

Supplementary Note **2**

Cochran-Armitage test of association 2

Allelic frequencies under a Causal Model 3

Estimation of Z Score for the causal model given by $\mathbf{W}$ and γ 6

Estimation of U_X, the covariance between G^X and Y, for the causal model given by $\mathbf{W}$ and γ 7

Estimation of V_X, the variance of G^X, for the causal model given by $\mathbf{W}$ and γ 7

Summary 10

Supplementary Tables **11**

Supplementary Figures **12**

Supplementary Note

Cochran-Armitage test of association

For a GWAS dataset, let $Y_i \in \{0,1\}$ denote the indicator of disease status at the ith sample. Let there be a total of N samples selected, with N_1 having been chosen from disease cases ($Y_i = 1$) and N_0 having been chosen from disease controls ($Y_i = 0$). Since this sampling is conditional upon case/control status, genotype frequencies may differ between our N samples and the whole population at disease associated SNPs. We therefore need to distinguish between which datasets the genotype probabilities are from; write $\mathbb{P}_{sam}$ for probabilities computed for the samples (i.e. $\mathbb{P}_{sam}(Y_i = 1) = \frac{N_1}{N}$), and $\mathbb{P}$ for probabilities generated with reference to the whole population.

Let n be the total number of SNPs. For any SNP X, write G_i^X for its genotype coding $\in \{0,1,2\}$ at sample i .

For the commonly used Cochran-Armitage test, the Z-Score at SNP X is computed as:

$$Z_X = \frac{U_X}{\sqrt{V}}$$

Where:

$$U_X = \sum_{i=1}^{N}((G_i^X - \overline{G^X})(Y_i - \bar{Y}))$$

$$V = (N-1)V_X V_Y$$

and V_X, V_Y are the variance of G^X and Y respectively:

$$V_X = \frac{N}{N-1}\frac{\sum_{i=1}^{N}(G_i^X - \overline{G^X})^2}{N}$$

$$V_Y = \frac{N_0 N_1}{N(N-1)}$$

i.e.:

$$V = \frac{N_0 N_1}{N(N-1)} \sum_{i=1}^{N} (G_i^X - \overline{G^X})^2$$

Under the null hypothesis of no association at SNP X, Z_X is distributed as a standard normal. Hence the two-sided p-value at X is given by:

$$p_X = 2(1 - \Phi(|Z_X|))$$

where Φ is the cumulative distribution function of the standard normal distribution. Conversely, given the unsigned p-value at X, the absolute value of the Z-Score is:

$$-\Phi^{-1}\left(\frac{p}{2}\right)$$

Allelic frequencies under a Causal Model

Write $\mathbf{W} = W_1, ..., W_m$ for the vector of causal SNPs. From phased publicly available reference datasets such as UK10K (**?**), it is possible to estimate haplotype frequencies across all SNPs in $\mathbf{W}$ at any subset of potential causal SNPs in control datasets. Since they are causal, these frequencies will differ in cases, and it is those frequencies we derive first. Note that, since sampling dependent only upon case/control status, we can assume:

$$\mathbb{P}_{sam}(G^{\mathbf{W}} = \mathbf{w}|Y = 0) = \mathbb{P}(G^{\mathbf{W}} = \mathbf{w}|Y = 0)$$

Write $\gamma_1, ..., \gamma_m$ for the log odds ratios of effect for the causal SNPs in the population. We assume that Y given $G^{\mathbf{W}}$ can be modelled as a binomial logistic regression. Then, from (**?**), the sample-specific odds ratios are the same as those at the population-level, and we can write:

$$\mathbb{P}_{sam}(Y_i = 1|G_i^{\mathbf{W}} = \mathbf{w}) = \frac{e^{\gamma_0 + \gamma_1 w_1 + ... + \gamma_m w_m}}{1 + e^{\gamma_0 + \gamma_1 w_1 + ... + \gamma_m w_m}}$$

where γ_0 is an intercept parameter. Since GWAS sampling is retrospective, the proportion of cases in the sample is fixed at $\frac{N_1}{N}$, constraining γ_0, which can be computed as follows:

$$\begin{aligned}
\mathbb{P}_{sam}(Y_i = 1) &= \frac{N_1}{N} \\
&= \sum_{\mathbf{w} \in \mathbb{Z}_3^m} \mathbb{P}_{sam}(Y_i = 1 | G_i^{\mathbf{W}} = \mathbf{w}) \mathbb{P}_{sam}(G_i^{\mathbf{W}} = \mathbf{w}) \\
&= \sum_{\mathbf{w} \in \mathbb{Z}_3^m} \mathbb{P}_{sam}(Y_i = 1 | G_i^{\mathbf{W}} = \mathbf{w}) \frac{\mathbb{P}_{sam}(Y_i = 0 | G_i^{\mathbf{W}} = \mathbf{w})}{\mathbb{P}_{sam}(Y_i = 0 | G_i^{\mathbf{W}} = \mathbf{w})} \mathbb{P}_{sam}(G_i^{\mathbf{W}} = \mathbf{w}) \\
&= \sum_{\mathbf{w} \in \mathbb{Z}_3^m} \frac{\mathbb{P}_{sam}(Y_i = 1 | G_i^{\mathbf{W}} = \mathbf{w})}{\mathbb{P}_{sam}(Y_i = 0 | G_i^{\mathbf{W}} = \mathbf{w})} \mathbb{P}_{sam}(Y_i = 0) \mathbb{P}(G_i^{\mathbf{W}} = \mathbf{w} | Y_i = 0) \\
&= \frac{N_0}{N} \sum_{\mathbf{w} \in \mathbb{Z}_3^m} e^{\gamma_0 + \gamma_1 w_1 + \ldots + \gamma_m w_m} \mathbb{P}(G_i^{\mathbf{W}} = \mathbf{w} | Y_i = 0)
\end{aligned}$$

$$\gamma_0 = \ln \left(\frac{N_1}{N_0 \sum_{\mathbf{w} \in \mathbb{Z}_3^m} e^{\gamma_1 w_1 + \ldots + \gamma_m w_m} \mathbb{P}(G_i^{\mathbf{W}} = \mathbf{w} | Y_i = 0)} \right)$$

Hence we can compute:

$$\mathbb{P}_{sam}(G_i^{\mathbf{W}} = \mathbf{w}) = \frac{\mathbb{P}(G_i^{\mathbf{W}} = \mathbf{w} | Y_i = 0) \mathbb{P}_{sam}(Y_i = 0)}{\mathbb{P}_{sam}(Y_i = 0 | G_i^{\mathbf{W}} = \mathbf{w})}$$

And also:

$$\mathbb{P}_{sam}(G_i^{\mathbf{W}} = \mathbf{w} | Y_i = 1) = \mathbb{P}(G_i^{\mathbf{W}} = \mathbf{w} | Y_i = 1) = \frac{\mathbb{P}_{sam}(Y_i = 1 | G_i^{\mathbf{W}} = \mathbf{w}) \mathbb{P}_{sam}(G_i^{\mathbf{W}} = \mathbf{w})}{\mathbb{P}_{sam}(Y_i = 1)}$$

To derive genotype probabilities at SNPs in LD with the causal SNPs, we assume that LD structures do not differ between cases and controls, and hence the correlation between $\mathbf{W}$ and X is independent of both disease status and our sampling. Thus:

$$\mathbb{P}_{sam}(G_i^X = x | G_i^{\mathbf{W}} = \mathbf{w}) = \mathbb{P}(G_i^X = x | G_i^{\mathbf{W}} = \mathbf{w})$$

and we can estimate, for both the whole population, and for our sample:

$$\mathbb{E}((G_i^X)^a|G_i^{\mathbf{W}}=\mathbf{w})=2^a\frac{\mathbb{P}(G_i^X=2\cap G_i^{\mathbf{W}}=\mathbf{w}|Y_i=0)}{\mathbb{P}(G_i^{\mathbf{W}}=\mathbf{w}|Y_i=0)}+\frac{\mathbb{P}(G_i^X=1\cap G_i^{\mathbf{W}}=\mathbf{w}|Y_i=0)}{\mathbb{P}(G_i^{\mathbf{W}}=\mathbf{w}|Y_i=0)}$$

from our reference dataset, for any constant a. From this, we compute:

$$\begin{aligned}\mathbb{E}((G_i^X)^a|Y_i=1) &= \sum_{\mathbf{w}\in\mathbb{Z}_3^m}\mathbb{E}((G_i^X)^a|G_i^{\mathbf{W}}=\mathbf{w})\mathbb{P}(G_i^{\mathbf{W}}=\mathbf{w}|Y_i=1)\\ &= \sum_{\mathbf{w}\in\mathbb{Z}_3^m}\frac{\mathbb{P}(G_i^{\mathbf{W}}=\mathbf{w}|Y_i=1)}{\mathbb{P}(G_i^{\mathbf{W}}=\mathbf{w}|Y_i=0)}\left[2^a\mathbb{P}(G_i^X=2\cap G_i^{\mathbf{W}}=\mathbf{w})+\mathbb{P}(G_i^X=1\cap G_i^{\mathbf{W}}=\mathbf{w})\right]\end{aligned}$$

$$\begin{aligned}\mathbb{E}((G_i^X)^a|Y_i=0) &= \sum_{\mathbf{w}\in\mathbb{Z}_3^m}\mathbb{E}((G_i^X)^a|G_i^{\mathbf{W}}=\mathbf{w})\mathbb{P}(G_i^{\mathbf{W}}=\mathbf{w}|Y_i=0)\\ &= \sum_{\mathbf{w}\in\mathbb{Z}_3^m}2^a\mathbb{P}(G_i^X=2\cap G_i^{\mathbf{W}}=\mathbf{w})+\mathbb{P}(G_i^X=1\cap G_i^{\mathbf{W}}=\mathbf{w})\end{aligned}$$

By expanding out the numerator in terms of probabilities within the sample dataset, we see that:

$$\begin{aligned}\frac{\mathbb{P}(G_i^{\mathbf{W}}=\mathbf{w}|Y_i=1)}{\mathbb{P}(G_i^{\mathbf{W}}=\mathbf{w}|Y_i=0)} &= \frac{\mathbb{P}_{sam}(Y_i=1|G_i^{\mathbf{W}}=\mathbf{w})\mathbb{P}_{sam}(G_i^{\mathbf{W}}=\mathbf{w})}{\mathbb{P}(G_i^{\mathbf{W}}=\mathbf{w}|Y_i=0)\mathbb{P}_{sam}(Y_i=1)}\\ &= \frac{\mathbb{P}_{sam}(Y_i=1|G_i^{\mathbf{W}}=\mathbf{w})\mathbb{P}_{sam}(G_i^{\mathbf{W}}=\mathbf{w}|Y_i=0)\mathbb{P}_{sam}(Y_i=0)}{\mathbb{P}(G_i^{\mathbf{W}}=\mathbf{w}|Y_i=0)\mathbb{P}_{sam}(Y_i=1)\mathbb{P}_{sam}(Y_i=0|G_i^{\mathbf{W}}=\mathbf{w})}\\ &= \frac{N_0}{N_1}e^{\gamma_0+\gamma_1 w_1+\ldots+\gamma_m w_m}\end{aligned}$$

And hence:

$$
\begin{aligned}
\mathbb{E}_{sam}((G_i^X)^a) &= \frac{N_1}{N}\mathbb{E}((G_i^X)^a|Y_i=1) + \frac{N_0}{N}\mathbb{E}((G_i^X)^a|Y_i=0) \\
&= \frac{N_0}{N}\sum_{\mathbf{w}\in\mathbb{Z}_3^m} e^{\gamma_0+\gamma_1 w_1+\ldots+\gamma_m w_m}\left[2^a\mathbb{P}(G_i^X=2\cap G_i^{\mathbf{W}}=\mathbf{w}) + \mathbb{P}(G_i^X=1\cap G_i^{\mathbf{W}}=\mathbf{w})\right] \\
&\quad + \frac{N_0}{N}\sum_{\mathbf{w}\in\mathbb{Z}_3^m}\left[2^a\mathbb{P}(G_i^X=2\cap G_i^{\mathbf{W}}=\mathbf{w}) + \mathbb{P}(G_i^X=1\cap G_i^{\mathbf{W}}=\mathbf{w})\right] \\
&= \frac{N_0}{N}\sum_{\mathbf{w}\in\mathbb{Z}_3^m}\left(e^{\gamma_0+\gamma_1 w_1+\ldots+\gamma_m w_m}+1\right) \\
&\qquad \left[2^a\mathbb{P}(G_i^X=2\cap G_i^{\mathbf{W}}=\mathbf{w}) + \mathbb{P}(G_i^X=1\cap G_i^{\mathbf{W}}=\mathbf{w})\right]
\end{aligned}
$$

Estimation of Z Score for the causal model given by W and γ

Finding the true expectation of $\frac{U_X}{\sqrt{V}}$ is intractable, so instead we compute a first order approximation by assuming independence:

$$\mathbb{E}(Z_X) = \mathbb{E}\left(\frac{U_X}{\sqrt{V}}\right) \approx \mathbb{E}(U_X) \times \mathbb{E}\left(\frac{1}{\sqrt{V}}\right)$$

These terms can be computed as shown in the following sections.

Estimation of U_X, the covariance between G^X and Y, for the causal model given by $\mathbf{W}$ and γ

We compute the expectation of U_X in our sample as follows:

$$\begin{aligned}
\mathbb{E}_{sam}(U_X) &= \mathbb{E}_{sam}\left[\sum_{i=1}^{N}(G_i^X - \overline{G^X})(Y_i - \bar{Y})\right] \\
&= \mathbb{E}_{sam}\left[N\left(\sum_{i=1}^{N} G_i^X Y_i\right) - \frac{1}{N}\left(\sum_{i=1}^{N} G_i^X\right)\left(\sum_{i=1}^{N} Y_i\right)\right] \\
&= N\mathbb{E}_{sam}(G_i^X Y_i) - \frac{1}{N}\left[N\mathbb{E}_{sam}(G_i^X Y_i) + N(N-1)\mathbb{E}_{sam}(G_i^X Y_j)\right] \qquad i \neq j \\
&= (N-1)\left[\mathbb{E}_{sam}(G_i^X Y_i) - \mathbb{E}_{sam}(G_i^X Y_j)\right] \\
&= (N-1)\left[\mathbb{E}_{sam}(G_i^X|Y_i = 1)\mathbb{P}_{sam}(Y_i = 1)\right] - \\
&\quad -(N-1)\mathbb{E}_{sam}(Y_j)\left[\mathbb{E}_{sam}(G_i^X|Y_i = 1)\mathbb{P}_{sam}(Y_i = 1) + \mathbb{E}_{sam}(G_i^X|Y_i = 0)\mathbb{P}_{sam}(Y_i = 0)\right] \\
&= \frac{(N-1)N_0 N_1}{N^2}\left[\mathbb{E}_{sam}(G_i^X|Y_i = 1) - \mathbb{E}_{sam}(G_i^X|Y_i = 0)\right]
\end{aligned}$$

Using the expressions for $\mathbb{E}_{sam}(G_i^X|Y_i)$ given in Section, this becomes:

$$\begin{aligned}
\mathbb{E}_{sam}(U_X) &= \frac{(N-1)N_0 N_1}{N^2}\sum_{\mathbf{w}\in\mathbb{Z}_3^m}\left[\left(\frac{N_0}{N_1}e^{\gamma_0+\gamma_1 w_1+\ldots+\gamma_m w_m} - 1\right)\right. \\
&\qquad \left.\left[2\mathbb{P}(G_i^X = 2 \cap G_i^{\mathbf{W}} = \mathbf{w}) + \mathbb{P}(G_i^X = 1 \cap G_i^{\mathbf{W}} = \mathbf{w})\right]\right]
\end{aligned}$$

Estimation of V_X, the variance of G^X, for the causal model given by $\mathbf{W}$ and γ

Recall:

$$\begin{aligned}
V_X &= \frac{1}{(N-1)}\sum_{i=1}^{N}(G_i^X - \overline{G^X})^2 \\
&= \frac{1}{(N-1)}\left[\left(\sum_{i=1}^{N}(G_i^X)^2\right) - \frac{1}{N}\left(\sum_{i=1}^{N} G_i^X\right)^2\right]
\end{aligned}$$

This is tractable, however, we need to find $\mathbb{E}\left(\frac{1}{\sqrt{V_X}}\right)$, which is more complex.

V_X is the variance of a normal, and so we model it as an Inverse Gamma (α, β) distribution. Then V_X^{-1} has a $\Gamma(\alpha, \beta^{-1})$ distribution, and $\sqrt{V_X^{-1}}$ has a generalised gamma distribution with parameters $p = 2, d = 2\alpha, a = \sqrt{\beta^{-1}}$. If $V_X \sim$ Inverse Gamma (α, β), then

$$\mathbb{E}(V_X) = \frac{\beta}{\alpha - 1} \qquad Var(V_X) = \frac{\beta^2}{(\alpha-1)^2(\alpha-2)}$$

Assuming we have computed $\mathbb{E}_{sam}(V_X)$ and $\mathbb{E}_{sam}(V_X^2)$, α and β are completely specified as:

$$\alpha = \frac{2\mathbb{E}(V_X^2) - (\mathbb{E}(V_X))^2}{\mathbb{E}(V_X^2) - (\mathbb{E}(V_X))^2} \qquad \beta = \frac{\mathbb{E}(V_X)\mathbb{E}(V_X^2)}{\mathbb{E}(V_X^2) - (\mathbb{E}(V_X))^2}$$

and $\mathbb{E}\left(\frac{1}{\sqrt{V_X}}\right)$ may be simply computed using:

$$\mathbb{E}\left(\frac{1}{\sqrt{V_X}}\right) = a\frac{\Gamma(\frac{d+1}{p})}{\Gamma(\frac{d}{p})} = \frac{1}{\sqrt{\beta}}\frac{\Gamma(\frac{2\alpha+1}{2})}{\Gamma(\alpha)}$$

Expectation of V_X

$$\begin{aligned}
\mathbb{E}_{sam}(V_X) &= \frac{1}{(N-1)}\left[N\mathbb{E}_{sam}((G_i^X)^2) - \frac{1}{N}\left(N\mathbb{E}_{sam}((G_i^X)^2) + N(N-1)\mathbb{E}_{sam}(G_i^X G_j^X)\right)\right] \\
&= \frac{1}{(N-1)}\left[(N-1)\mathbb{E}_{sam}((G_i^X)^2) - (N-1)\mathbb{E}_{sam}(G_i^X G_j^X)\right] \\
&= \mathbb{E}_{sam}((G_i^X)^2) - (\mathbb{E}_{sam}(G_i^X))^2
\end{aligned}$$

Expectation of V_X^2

$$\mathbb{E}_{sam}(V_X^2) = \left(\frac{1}{(N-1)}\right)^2 \mathbb{E}_{sam}\left[\left(\sum_{i=1}^N (G_i^X)^2\right)^2 - \frac{2}{N}\left(\sum_{i=1}^N (G_i^X)^2\right)\left(\sum_{i=1}^N G_i^X\right)^2 + \frac{1}{N^2}\left(\sum_{i=1}^N G_i^X\right)^4\right]$$

Let $E_n = \mathbb{E}_{sam}((G_i^X)^n)$. Breaking this down into terms, for (i, j, k, l) representing different

indices, we have:

$$\begin{aligned}
& \mathbb{E}_{sam}\left[\left(\sum_{i=1}^{N}(G_i^X)^2\right)^2\right] \\
= \;& N\mathbb{E}_{sam}((G_i^X)^4) + N(N-1)\mathbb{E}_{sam}((G_i^X)^2(G_j^X)^2) \\
= \;& NE_4 + N(N-1)
\end{aligned}$$

$$\begin{aligned}
& \mathbb{E}_{sam}\left[\left(\sum_{i=1}^{N}(G_i^X)^2\right)\left(\sum_{i=1}^{N}G_i^X\right)^2\right] \\
= \;& N\mathbb{E}_{sam}((G_i^X)^4) + 2N(N-1)\mathbb{E}_{sam}((G_i^X)^3(G_j^X)) + N(N-1)\mathbb{E}_{sam}((G_i^X)^2(G_j^X)^2) + \\
+ \;& N(N-1)(N-2)\mathbb{E}_{sam}((G_i^X)^2(G_j^X)(G_k^X)) \\
= \;& NE_4 + 2N(N-2)E_3E_1 + N(N-1)E_2^2 + N(N-1)(N-2)E_2E_1^2
\end{aligned}$$

$$\begin{aligned}
& \mathbb{E}_{sam}\left[\left(\sum_{i=1}^{N}G_i^X\right)^4\right] \\
= \;& N\mathbb{E}_{sam}((G_i^X)^4) + 4N(N-1)\mathbb{E}_{sam}((G_i^X)^3(G_j^X)) + 6N(N-1)\mathbb{E}_{sam}((G_i^X)^2(G_j^X)^2) + \\
+ \;& 6N(N-1)(N-2)\mathbb{E}_{sam}((G_i^X)^2(G_j^X)(G_k^X)) + \\
+ \;& N(N-1)(N-2)(N-3)\mathbb{E}_{sam}((G_i^X)(G_j^X)(G_k^X)(G_l^X)) \\
= \;& NE_4 + 4N(N-1)E_3E_1 + 6N(N-1)E_2^2 + 6N(N-1)(N-2)E_2E_1^2 + \\
+ \;& N(N-1)(N-2)(N-3)E_1^4
\end{aligned}$$

Giving:

$$\mathbb{E}_{sam}(V_X^2) = \frac{1}{N}E_4 - \frac{4}{N}E_3E_1 + 2\frac{N^2-2N+6}{N(N-1)}E_2^2 - 2\frac{(N-2)(N-3)}{N(N-1)}E_2E_1^2 + \frac{(N-2)(N-3)}{N(N-1)}E_1^4$$

Summary

Thus, given only a choice of which SNPs are causal ($\mathbf{W}$), their effect sizes ($\boldsymbol{\gamma}$), sample sizes (N_0, N_1) and a reference dataset from which we can derive allele frequencies ($\mathbb{E}(G_i^X | Y_i = 0)$) and the relationships between SNPs ($\mathbb{E}(G_i^X | G_i^{\mathbf{W}} = \mathbf{w})$), we can derive an expected Z Score, $\mathbf{Z}^{EXP}$ at any SNP, causal or not.

This can then be used directly. However, most applications require simulated output from such a GWAS. $\mathbf{Z}^{SIM}$ can therefore be computed, which will be distributed:

$$\mathbf{Z}^{SIM} \sim \mathbf{N}(\mathbf{Z}^{EXP}, \boldsymbol{\Sigma})$$

where $\boldsymbol{\Sigma}$ is the genotype correlation matrix for the SNPs in this region (**?**).

Supplementary Tables

Table S1 (file: simgwas-supptab.csv): Comparison of beta (log OR) and Z scores from simulations run with simGWAS, HapGen, and forward simulation. Mean and standard deviations are given at causal variants and one unlinked variant per scenario. Means were compared using T tests with Welch extension to accommodate unequal standard deviations, and p values are shown in the columns ".meantest". Formal comparison of the full distribution was conducted by Kolmogorov-Smirnov tests (KS) and p values are shown in the columns ".KStest". The sample size (n) is the number of cases and controls - i.e. n=1000 indicates the simulations related to 1000 cases and 1000 controls. Each plot summarises 1000 simulations. The scenario label gives the corresponding "scenario-snp" pair - i.e. the label 3-1 refers to scenario 3, first causal SNP. Unlinked variants are denoted as SNP 0.

Supplementary Figures

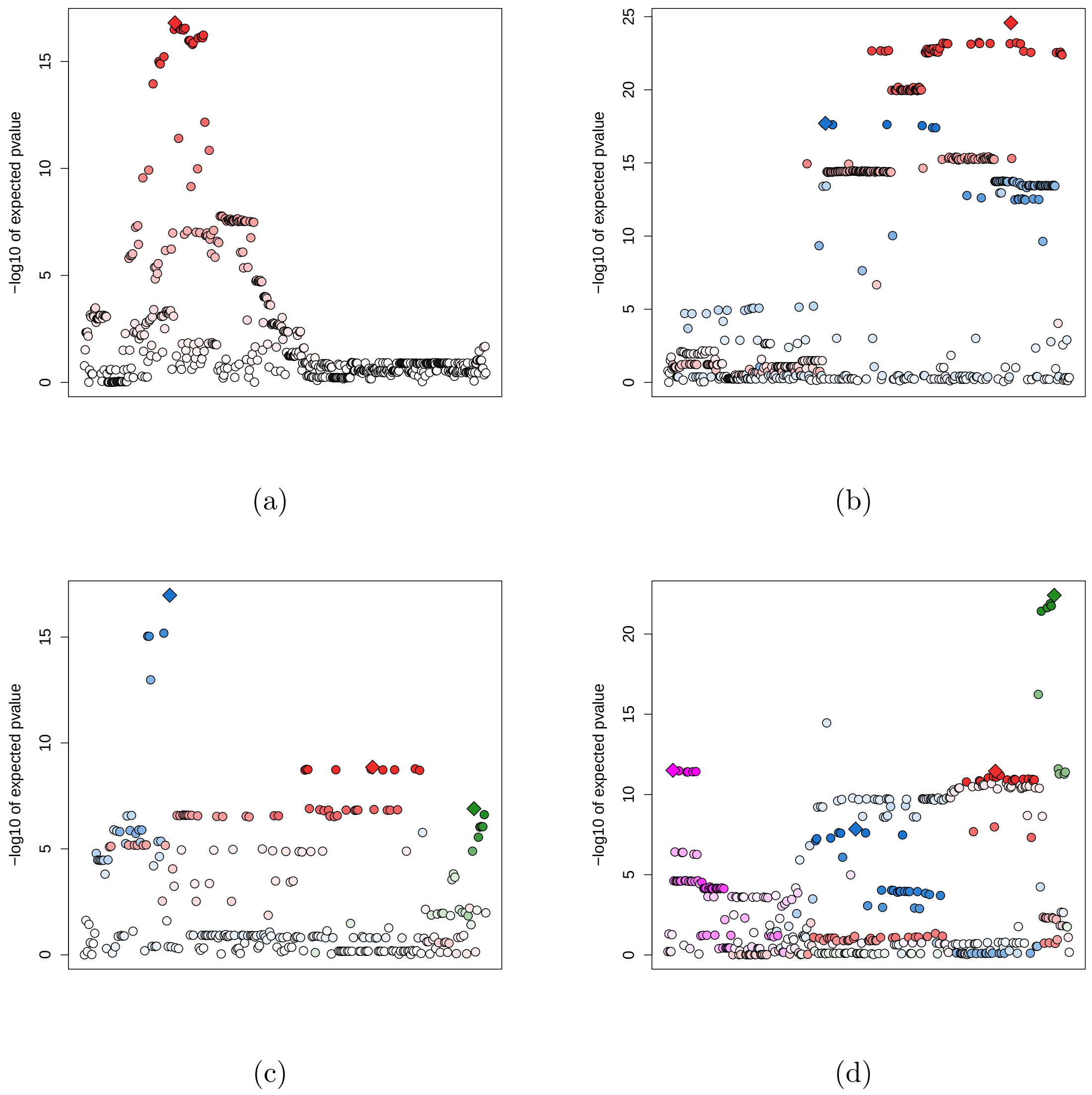

Figure S1: Local Manhattan plots for p values generated from expected Z scores under different scenarios, in order to confirm by visual inspection that the expected Z Scores produced by our algorithm are consistent with the behaviour we would expect from their causal SNPs. In order to easily see the pattern of association, causal variants chosen were common, with a strong effect and (in the case of multiple causal variants) only weakly linked. Causal SNPs are designated by a coloured diamond. Non-causal SNPs are designated by a circle, coloured according to their LD with their most correlated causal SNP. In each scenario, 5000 cases and 5000 controls were simulated. (a) A single causal variant with MAF $= 0.34$ and Odds Ratio of effect $= 1.3$ (b) Two causal variants with MAF $= (0.14, 0.30)$ and Odds Ratio of effect $= (1.5, 1.2)$ (c) Three causal variants with MAF $= (0.12, 0.43, 0.17)$ and Odds Ratio of effect $= (1.2, 1.2, 1.2)$ (d) Four causal variants with MAF $= (0.33, 0.44, 0.17, 0.28)$ and Odds Ratio of effect $= (1.5, 1.5, 1.5, 1.5)$

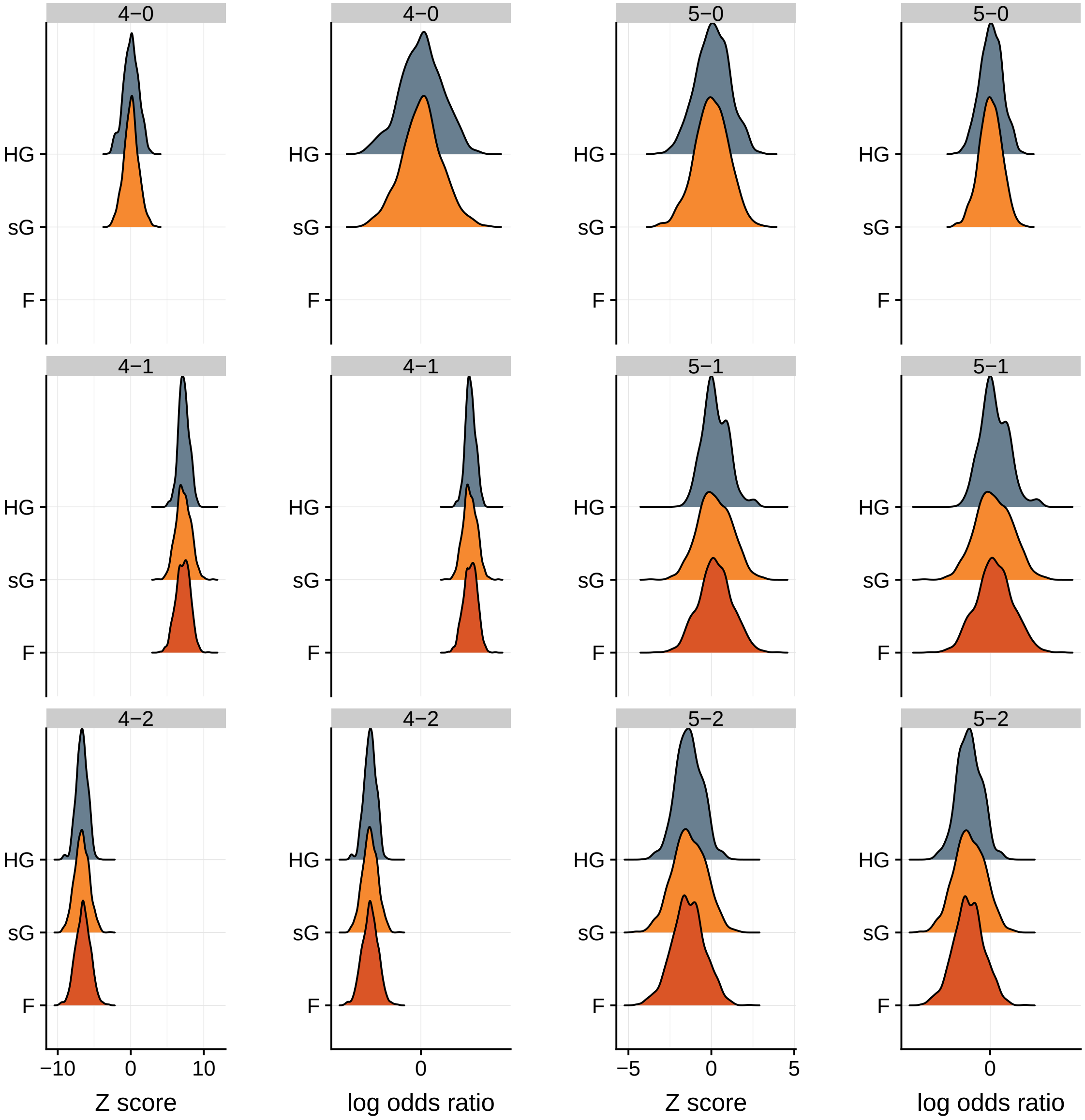

Figure S2: Results from simGWAS (sG) are similar to those from HAPGEN+SNPTEST (HG) and forward simulation (F). Distributions of simulated Z scores and log odds ratios are shown from 1000 simulations assuming 5000 cases and 5000 controls under two scenarios - 4 and 5 - described in Table **??**. The label of each plot gives the corresponding scenario-SNP pair, with SNP 0 unlinked ($r^2 < 0.15$) to either causal variants (no forward simulation results for SNP 0). In scenarios 4 and 5, two SNPs have the same effect sizes but are either weakly linked ($r = 0.15$, scenario 4) or in strong LD ($r = 0.8$, scenario 5). Note that the marginal effect sizes are closer to 0 in scenario 5 because the linked effects cancel when considering marginal association.
